# Complementary therapy in Chinese medicine for dilated cardiomyopathy with congestive heart failure: A three-year follow-up case report

**DOI:** 10.1097/MD.0000000000042389

**Published:** 2025-05-16

**Authors:** Huiyan Feng, Xinyu Liu, Rui Shi, Yazhi Xi, Yue Deng

**Affiliations:** a Department of Cardiovascular Medicine, Changchun University of Chinese Medicine, Changchun, China; b Department of Cardiovascular Medicine, Shanghai 411 Hospital, Shanghai, China; c Department of Cardiovascular Medicine, Affiliated Hospital, Changchun University of Chinese Medicine, Changchun, China; d TCM Cardiovascular Clinical Medicine Research Center of Jilin Province, Changchun, China.

**Keywords:** case report, congestive heart failure, dilated cardiomyopathy, traditional Chinese medicine

## Abstract

**Rationale:**

Dilated cardiomyopathy (DCM) coupled with congestive heart failure (CHF) is a clinically refractory cardiovascular condition characterized by high hospitalization and death rates. This case report presents a new treatment with traditional Chinese medicine (TCM). The patient’s condition was improved through the Yi-Qi-Hua-Yu-Li-Shui decoction which provides ideas for other patients.

**Patient concerns:**

A 26-year-old patient presented with dyspnea and recurrent chest tightness following exertion. Echocardiogram revealed a left ventricular end-diastolic dimension of 73 mm and a left ventricular ejection fraction of 31%. N-terminal pro-B-type natriuretic peptide was 2679 pg/mL.

**Diagnoses:**

The patient was diagnosed with DCM and CHF.

**Interventions:**

The patient used vasodilators, diuretics, cardiac stimulants, and many others, but the results were not encouraging. Consequently, he sought further intervention through the TCM. The principle of TCM involves benefiting qi, promoting blood circulation, resolving blood stasis, and inducing diuresis through oral Chinese herbal soup.

**Outcomes:**

The patient’s clinical symptoms were greatly reduced after receiving TCM treatment for 15 days. After 10 months, cardiac function returned to normal with a left ventricular ejection fraction of 58% and left ventricular end-diastolic dimension of 54 mm, N-terminal pro-B-type natriuretic peptide decreased from 2679 pg/mL to 440 pg/mL. Following more than 3 years of observation, the patient had no rehospitalization and led a mostly normal life.

**Lessons:**

In the successful therapy of DCM-CHF, we discovered that Yi-Qi-Hua-Yu-Li-Shui decoction can significantly improve cardiac function, improve exercise tolerance, reduce clinical symptoms, and lower the rate of readmission.

## 1. Introduction

Systolic insufficiency and dilation of the left or both ventricles are the hallmarks of dilated cardiomyopathy (DCM), which ultimately results in heart failure (HF). For left ventricular ejection fraction (LVEF) <35%, guidelines advise heart transplantation or the implantation of a cardioverter defibrillator to prevent sudden cardiac death.^[1]^ More than 64 million people worldwide suffer from HF, which has become a serious public health issue with a 25% 5-year survival rate after hospitalization.^[2,3]^ After 12.7 years of observation, a study of the age-dependent correlation of risk factors revealed that old age was associated with a greater prevalence of HF and younger patients with hypertension were associated with a 3-fold increased risk of future HF (hazard ratio [HR] = 3.02;95% confidence interval [CI]: 2.10–4.34; *P* ＜ .001).^[4]^ Although there have been new guidelines for the care of HF in recent years, there is a paucity of evidence-based strategies to improve prognosis and lower mortality, and treatment outcomes vary widely.^[5,6]^ In China, the mortality rate among HF patients is 13.7% at 1 year and 28.2% at 3 years after hospital discharge, indicating suboptimal adherence to guideline-directed medical therapy (GDMT).^[7]^ Studies have demonstrated that under GDMT, the all-cause mortality rates for DCM are 55.9% at 5 years and 65.8% at 15 years, with LVEF ≤ 35% identified as an independent risk factor.^[8]^

**Figure 1.**
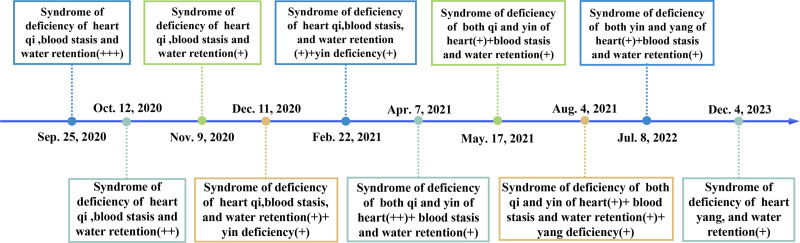
The timeline of the medication process and changes in the syndrome of the patient. (+++): severe; (++): moderate; (+): mild.

**Figure 2.**
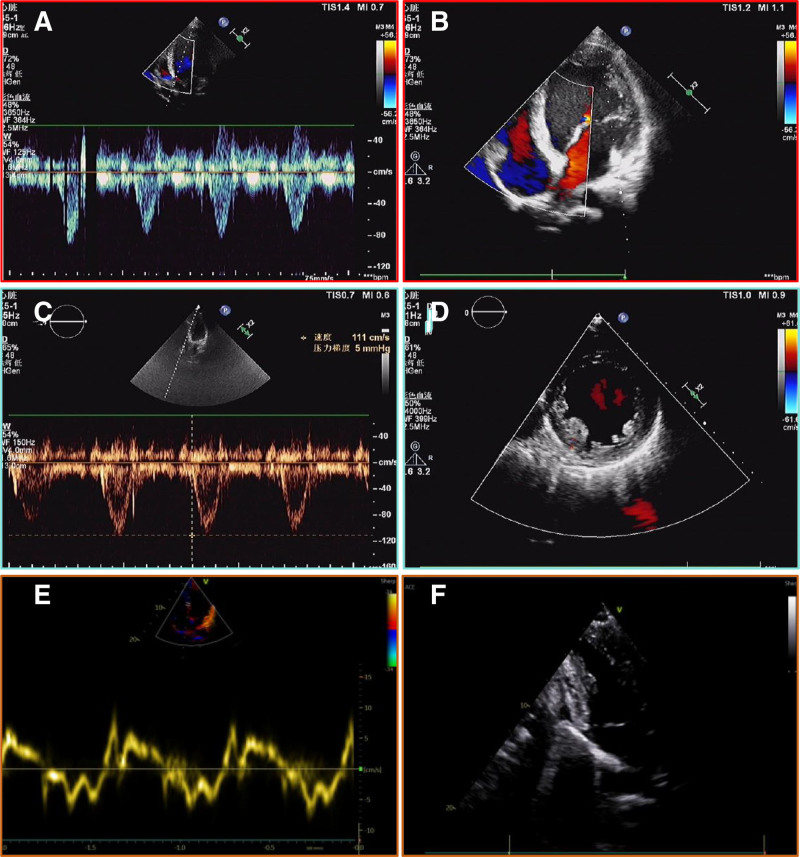
Echocardiogram images at different times. (A, B) on Nov.9, 2020, decreased centripetal motion of the walls of the left ventricle and uncoordinated ventricular wall motion, enlarged left heart, regurgitation at all valves, reduced systolic function; pericardial effusion. (C, D) On Feb.22, 2021: left ventricular myocardial changes; enlarged left heart; trace regurgitation of the bicuspid and tricuspid valves; reduced left ventricular systolic and diastolic function; trace pericardial cavity effusion. (E, F) on Dec 4, 2023: no abnormalities in left ventricular systolic and diastolic function.

**Figure 3.**
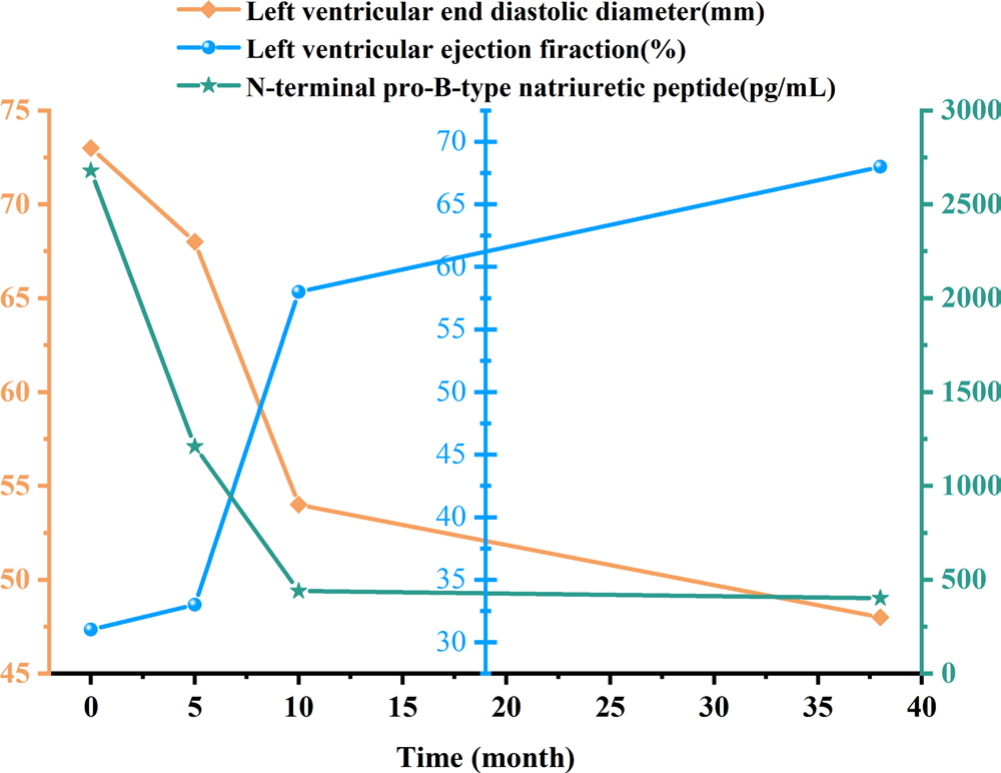
Changes in cardiac function parameters of admission and follow-up. LVEDD = left ventricular end diastolic diameter, LVEF = left ventricular ejection firaction, NT-proBNP = N-terminal pro-B-type natriuretic peptide.

TCM is known for being low-cost and having minimal harmful effects. According to a meta-analysis, treating DCM-HF with 5 TCM injections in addition to western medicine is significantly more effective than using western medicine alone.^[9]^ Chinese herbal remedies have been shown in pharmacological investigations to enhance cardiomyocyte energy metabolism, which is beneficial for HF.^[10]^ However, there is a dearth of information regarding oral Chinese herbal remedies for DCM-HF. Here, we report for the first time on the effective use of oral Yi-Qi-Hua-Yu-Li-Shui decoction (YQHYLSD) in conjunction with conventional western medicine, which helped the patient avoid surgery-related anxiety and lower costs. The syndrome is a comprehensive summary of the pathology of a particular stage or kind of disease process (Fig. 1). It highlights the dynamic nature of the disease and how it evolves over time. We evaluated the efficacy of treatment using noninvasive and visual methods, such as echocardiogram (ECHO) and biomarker (Table 1, Fig. 2 and 3). Those records are also necessary, such as the Zheng score, Minnesota Living with Heart Failure Questionnaire (MLHFQ) score, and 6-minute walk test (see Table 2, Table 1 and 2, Supplemental Table 2, Supplemental Digital Content, https://links.lww.com/MD/O896, which demonstrates the improvement of the Zheng score, cardiac function and quality of life.). This study provides clinical experience with the application of TCM.

**Table 1:** Changes in cardiac function parameters of admission and follow-up.

Time	LVEDD (mm)	LVEF (%)	E/A	NYHA Class	NT-proBNP (pg/mL)
Admission	73	31	-	IV	2679
1.5 month	74	28	-	III	-
5 month	68	33	<1	II	1210
10 month	54	58	>1	I	440
21.5 month	50	70	>1	I	-
38 month	48	68	>1	I	402

LVEDD = left ventricular end diastolic diameter, LVEF = left ventricular ejection fraction, NT-proBNP = N-terminal pro-B-type natriuretic peptide, NYHA = New York Heart Association.

**Table 2:** Dynamic trajectories of health-related quality of life during treatment.

Date	HR (beats/min)	BP (mm Hg)	Syndrome score	6MWT (m)	MLHFQ
Sep. 25, 2020	102	90/66	80	100	105
Oct. 12, 2020	89	95/68	72	200	95
Nov. 9, 2020	65	101/67	56	300	88
Dec. 11, 2020	68	103/71	40	400	67
Feb. 22, 2021	64	105/72	32	470	50
Apr. 7, 2021	65	106/74	28	500	46
May. 17, 2021	66	106/77	24	710	41
Aug. 4, 2021	67	109/77	20	780	32
July. 8, 2022	65	110/75	20	790	20
Dec. 4, 2023	65	112/76	14	800	11

6MWT = 6-minute walk test, BP = blood pressure, HR = heart rate, MLHFQ = Minnesota Living with Heart Failure Questionnaire.

## 2. Case presentation

### 2.1. Clinical information

A 26-year-old man sought TCM treatment for “shortness of breath following exertion for 3 years, aggravated for 7 days,” and went to the Department of Cardiology of Affiliated Hospital of Changchun University of Chinese Medicine (CCUCM) on September 25, 2020. Three years ago, he was brought to Dongliao County People’s Hospital with paroxysmal chest tightness and dyspnea for many hours, which were alleviated by sitting for 30 minutes. ECHO examination revealed that left ventricular end-diastolic dimension (LVEDD) was 72 mm and LVEF was 28%, N-terminal pro-B-type natriuretic peptide (NT-proBNP) was 1198 pg/mL, and the patient was diagnosed with HF. After the condition improved, the patient was released from the hospital. The symptoms returned and grew worse over time. He sought advice from nearby hospitals and Jilin University’s First Hospital of Baiqiu’en, but the results were not encouraging. The patient denied having ever experienced a brain hemorrhage, cerebral infarction, or allergy, but reported a 5-year history of hypertension, with a maximum reading of 180/110 mm Hg. The patient’s father suffered from HF and hypertension for a long time. ECHO (Sept. 13, 2020, Affiliated Hospital, CCUCM): left atrium: 52 mm, right ventricle: 25 mm, LVEDD: 73 mm, right atrium: 57 × 47 mm, LVEF: 31% (Table 1); left ventricular myocardial changes, left heart and right atrial enlargement; moderate mitral regurgitation; moderate-severe tricuspid regurgitation; mild aortic regurgitation; trace pulmonary regurgitation; decreased left ventricular systolic function; pericardial effusion. NT-proBNP was 2679 pg/mL. Physical examination noted bilateral lower extremity edema, wet rales audible in both lungs, a displaced apical beat 1 cm to the left, an enlarged heart with relative dullness, a heart rate (HR) of 102 beats/min, arrhythmia, heart sounds of varying intensity, and a blood pressure (BP) of 90/66 mm Hg. The patient received prescribed diuretics, cardiotonic drugs, and other medications, yet the treatment response was poor. To sum up, based on the patient’s medical history and ECHO examination findings (LVEDD: 73 mm, LVEF: 31%), the patient was diagnosed with HF (New York Heart Association, NYHA, class IV) and DCM.

### 2.2. TCM therapy

Based on the 4 diagnostic methods of TCM (looking, listening, asking, and feeling the pulse), the clinical symptoms included shortness of breath following exertion, panic attacks, wheezing and difficulty falling asleep, chest pain and tightness, heavy sweating, decreased urine output, and distended stools that occurred once every 3 days; the tongue seemed pale and enlarged, with a greasy coating; and the pulse was empty. TCM syndrome was differentiated as heart qi deficiency, blood stasis, and water stagnation. The principle of TCM involves benefiting qi, promoting blood circulation, resolving blood stasis and inducing diuresis through oral Chinese herbal soup: Huang Qi 80 g, Ren Shen10 g, Hong Jing Tian 12 g, Dan Shen 10 g, Ting Li Zi 20 g, Zi Qiao 10 g, Jiang Ban Xia 10 g, Fu Ling 18 g, Tu Fu Ling 30 g, Bai Mao Gen 20 g, Qing Hao 18 g, Gui Jia 12 g, Gan Song 12 g, Jiang Xiang 10 g. Further adjustments during follow-up consultations are all shown in Table 3. Chinese herbal drinks are prepared by soaking 450 mL of water for an hour, then decocting for 30 min on a gentle fire, and taking 150 mL orally after breakfast and dinner for 15 days. Advise the patient to follow up if they have any adverse reactions after the medication. Chinese herbal medicines are supplied by the Chinese herbal pharmacy of the Affiliated Hospital of CCUCM, and prepared by the Decoction Center according to the established process.

**Table 3:** Changes in condition and medication adjustments during treatment.

Date	Patient symptoms	TCM
Sep. 25, 2020	Shortness of breath following exertion, decreased urine output, chest pain, pale and enlarged tongue, coating greasy, empty pulse, and et al.	Huang Qi 80 g, Ren Shen10 g, Hong Jing Tian 12 g, Dan Shen 10 g, Ting Li Zi 20 g, Zi Qiao 10 g, Jiang Ban Xia 10 g, Fu Ling 18 g, Tu Fu Ling 30 g, Bai Mao Gen 20 g, Qing Hao 18 g, Gui Jia 12 g, Gan Song 12 g, Jiang Xiang 10 g; 15 days
Oct. 12, 2020	The symptoms were relieved.	As last; 21 days
Nov. 9, 2020	The symptoms were relieved, but poor sleep, and et al.	Add Long Gu 30 g, Mu Li 30 g,Wu Wei Zi 6 g, Suan Zao Ren 10 g, Ci Shi 18 g; 30 days
Dec. 11, 2020	The symptoms were stable, less coated, rapid pulse, and et al.	Remove Jiang Ban Xia, Fu Ling and add Mai Dong 12 g, Sheng Di Huang12 g; 15 days
Feb. 22, 2021	All symptoms were improved, vomiting, and peeling moss, and et al.	Add Xuan Fu Hua 10 g, Dai Zhe Shi 12 g, Chen Pi 10 g; 21 days
Apr. 7, 2021	Activity as usual, dry mouth, pale and cracked tongue, white coating, relaxed pulse, and et al.	As YQHYLSD, remove Hong Jing Tian, Jiang Ban Xia, Fu Ling, Bai Mao Gen, Qing Hao and Gui Jia, add Mai Dong 20 g, Wu Wei Zi 10 g, Zhi Mu 10 g, Dan Pi 10g; 21 days
May 17, 2021	Exfoliatoid coating, relaxed pulse, and et al.	Add Yu Zhu 6 g, Jiang Ban Xia 10 g, Chen Pi 6 g; 21 days
Aug. 4, 2021	Symptoms were disappeared, but daytime sweating	Add Bai Zhu 10 g, Fang Feng 12 g, Xian He Cao 20 g; 21 days
Jul. 8, 2022	Enlarged tongue, white and greasy coating, empty and rapid pulse, and et al.	Add Ze Xie 10 g; 21 days
Dec. 4, 2023	Purple tongue, thick and exfoliative coating, deep and relaxed pulse, and et, al.	Qi Li Qiang Xin Capsules;21 days

The prescription modifications, encompassing herb additions and subtractions, are executed proportionally to the quantified changes in four-diagnostic indices and the transition of TCM syndrome differentiations.

### 2.3. Treatment outcomes

The patient’s clinical symptoms were greatly reduced after TCM treatment. From Table 1, cardiac function reverted to normal with an LVEF of 58% and LVEDD of 54 mm after 10.5 months; 21.5 months later, the LVEF was 70% and the LVEDD was 50 mm; NT-proBNP decreased from 2679 pg/mL to 402 pg/mL; the NYHA classification decreased from IV to I. Meanwhile, the syndrome score decreased from 80 to 12, the MLHFQ score from 105 to 11, and the 6-minute walk test increased from 100 to 800 meters (see Table 2, Table 1 and 2, Supplemental Digital Content, https://links.lww.com/MD/O896), all of which demonstrated the efficacy of TCM. Regular telephone and clinical follow-ups and test indicators such as blood counts and liver and kidney functions showed that the patient had no adverse reactions during drug administration except for occasional gastrointestinal discomforts, which disappeared after discontinuation of the drug.

## 3. Discussion

Historically, most reported cases of DCM-HF involved pediatric populations, lacked integration of TCM diagnostic or therapeutic approaches, and were characterized by limited or absent long-term follow-up data.^[11–14]^ While HF predominantly affects individuals over 65 years old, studies by Nedkoff L et al highlight a rising trend in hospital admissions for HF among younger adults.^[15,16]^ A French cohort study of individuals aged 18–50 years reported an HF incidence of 4.7%, representing a statistically significant increase of 0.041% (*P* ＜ .001), with poor 2-year outcomes (eg, high mortality and rehospitalization rates) linked to obesity, smoking, hypertension, dyslipidemia, and diabetes mellitus.^[17]^ The patient is a 26-year-old male with a 5-year history of hypertension and a 3-year history of HF. The father had a long history of HF and hypertension. We speculate that the etiology in this case might be related to inadequate hypertension control or a familial genetic susceptibility in the family. Given that heredity plays a significant role in DCM, clinical decision-making for HF can be improved with the application of genetic testing. Currently, extensive research is being done on risk markers.^[18]^ Over a period of more than 3 years, we accurately and thoroughly documented the patient’s entire journey, which included cardiac enlargement, poor quality of life and management, recurrent episodes of illness, and abnormal systolic and diastolic function. Following TCM treatment, the patient’s symptoms essentially disappeared, the quality of life was maintained, and the cardiac function recovered. With LVEF and chamber size changes serving as the primary markers, ECHO can be used to diagnose and assess treatment outcomes for patients with HF. It offers a thorough, noninvasive assessment of the patient’s heart anatomy and function.^[19,20]^

Rooted in the *Huangdi Neijing* (Yellow Emperor’s Inner Canon), a foundational text of TCM with millennia of history, TCM offers early documented descriptions of HF and DCM. TCM holds that the 2 fundamental disorders of DCM-HF are deficiency of qi and blood stasis, and key elements in the pathophysiology of both are phlegm-dampness. At the beginning, it manifests as qi deficiency, gradually progressing to deficiency of either yin or yang, and ultimately depletion of yin causing collapse of yang (Fig. 1). According to the principle of benefiting qi, resolving blood stasis, and promoting diuresis, we composed an empirical recipe: YQHYLSD (Table 3). The formula embodies TCM’s holistic approach, addressing both physical and mental health, harmonizing yin and yang, and targeting both symptoms and root causes. Huang Qi (Astragalus membranaceus), a core component of YQHYLSD, contains astragaloside IV, which demonstrates cardioprotective effects in HF by mitigating myocardial ischemia, modulating sarcoplasmic reticulum Ca²⁺-ATPase activity, enhance energy metabolism, encourage neovascularization, prevent myocardial hypertrophy and fibrosis, and lower cardiomyocyte apoptosis in HF.^[21]^ high-dose Huang Qi combined with Ren Shen (Panax ginseng) can improve myocardial remodeling and contractility, which enhances cardiac function. The mechanism governing these cardiovascular effects may be linked to oxidative stress or the control of cardiomyocyte apoptosis.^[22]^ Chest tightness and pain symptoms can be alleviated by stimulating blood circulation with the help of Dan Shen (Salvia miltiorrhiza) and Chuan Xiong (Ligusticum chuanxiong). It has been demonstrated that the principal active ingredients of Ren Shen and Dan Shen function as Na⁺/K⁺-ATPase inhibitors and can cure cardiovascular illness by using a similar molecular mechanism to cardiac glycosides.^[23]^ Bai Mao Gen (Imperata cylindrica), Ting Li Zi (Lepidium apetalum), and Tu Fu Ling (Smilax glabra) alleviated lung congestion, increased urine output, and improved edema, dyspnea, and orthopnea by targeting the lung and bladder meridians. In addition to regulating the spleen and stomach and improving tongue appearance, Fu Ling (Poria cocos), Jiang Ban Xia (Pinellia ternata), and Zhi Qiao (Citrus aurantium) can also strengthen Huang Qi’s diuresis-inducing and qi-tonifying effects. In addition to improving sleeplessness, panic attacks, excessive perspiration, dry mouth, and Qing Hao (Artemisia annua) and Gui Jia (Plastrum testudinis) also help normalize blood pressure and control heart rate. Jiang Xiang (Dalbergia odorifera) and Gan Song (Nardostachys jatamansi) harmonize qi flow, easing anxiety and depressive symptoms. The formula exemplifies the holism of TCM wonderfully; it treats the body and mind simultaneously, balances yin and yang, and treats both the symptoms and the underlying cause.

Qi Li Qiang Xin is a cardiotonic herbal formulation that has been incorporated into Chinese medicine guidelines for the therapy of heart failure.^[24,25]^ Changes in lifestyle and appropriate exercise also contribute to improve heart function in HF patients. According to a meta-analysis, TCM techniques including Ba duan jin (Eight-Section Brocade) and Tai ji quan (Tai Chi) may help improve symptoms and clinical outcomes in HF patients.^[26]^ Prabhu S et al showed that yoga can be utilized as an adjuvant to help improve ECHO parameters in patients with lower NYHA class or HF with reduced ejection fraction, thereby improving quality of life and prognosis.^[27]^

## 4. Conclusion

YQHYLSD enhanced cardiac function, reverse ventricular remodeling and improve quality of life of the patient with DCM-HF. This case report provides cardiologists with therapeutic ideas and suggestions, but in clinical practice, it should be considered according to the different conditions of different patients. Moreover, the mechanism of YQHYLSD needs further research and exploration. Clinical randomized controlled trials and animal studies will be carried out next.

## Author contributions

**Conceptualization:** Huiyan Feng, Yazhi Xi, Yue Deng.

**Data curation:** Huiyan Feng.

**Visualization:** Huiyan Feng.

**Writing – original draft:** Huiyan Feng, Xinyu Liu.

**Writing – review & editing:** Huiyan Feng.

**Methodology:** Xinyu Liu.

**Resources:** Xinyu Liu, Rui Shi, Yazhi Xi, Yue Deng.

**Funding acquisition:** Rui Shi, Yue Deng.

**Investigation:** Rui Shi.

**Project administration:** Yue Deng.

**Supervision:** Yue Deng.

## Supplementary Material
