# Supplementary material for: Complementary therapy in Chinese medicine for dilated cardiomyopathy with congestive heart failure: A three-year follow-up case report
Source: Medicine (Baltimore). 2025 May 16;104(20):e42389. doi: 10.1097/MD.0000000000042389 (PMC12091609; doi:10.1097/MD.0000000000042389)
Supplement: Supplementary file 1 [file medi-104-e42389-s001.pdf]

Supplemental Table 1 Minnesota Living with Heart Failure Questionnaire Scales during treatment.

Serial	Observation Items	Sep. 25, 2020	Oct. 12, 2020	Nov. 9, 2020	Dec. 11, 2020	Feb. 22, 2021	Apr. 7, 2021	May.17, 2021	Aug. 4, 2021	July. 8, 2022	Dec. 4, 2023
1	Swelling in your ankles or legs?	5	4	3	2	2	2	2	1	1	0
2	Forced you to sit or lie down during the day?	5	4	3	2	2	2	2	1	1	0
3	Made walking or climbing stairs difficult?	5	4	3	3	2	2	2	2	1	1
4	Made housework or yard work difficult?	5	4	3	2	2	2	2	2	1	1
5	Made leaving home difficult?	5	4	3	3	2	2	2	2	1	0
6	Disrupted your sleep at night?	5	5	5	3	2	1	1	1	1	0
7	Made doing things with friends/family difficult?	5	4	4	2	2	2	2	1	1	1
8	Affected your ability to work for income?	5	4	4	3	2	2	2	2	2	1
9	Hindered recreational activities or hobbies?	5	5	5	4	3	2	2	2	2	1
10	Affected your sexual life?	5	5	5	4	3	2	2	2	0	1
11	Reduced your appetite for favorite foods?	5	4	5	4	2	2	1	1	1	0
12	Caused shortness of breath?	5	5	4	3	2	2	2	2	1	0
13	Caused fatigue, weakness, or	5	5	5	4	3	3	2	2	1	1

	lack of energy?										
14	Led to hospitalization?	5	4	3	2	2	2	2	2	1	0
15	Caused financial burden from medical expenses?	5	5	4	3	3	3	3	2	1	1
16	Caused side effects from treatment?	5	4	4	3	2	2	2	1	1	0
17	Made you feel like a burden to family/friends?	5	5	5	4	3	3	2	2	1	1
18	Made you feel a loss of control over your life?	5	5	5	4	2	2	2	1	0	0
19	Caused worry?	5	5	5	4	3	3	2	2	1	1
20	Affected concentration or memory?	5	5	5	4	3	2	2	1	1	1
21	Caused low mood or depression?	5	5	5	4	3	3	2	1	0	0
	Total Score	105	95	88	67	50	46	41	33	20	11

Note: How has your heart failure affected your life in the past month (4 weeks)? None is 0, very mild is 1,mild is 2, moderate is 3, severe is 4, and very severe is 5. The lower the score, the better the treatment efficacy and the higher the quality of life.

Supplemental Table 2 Chinese Medicine Syndrome Scales during treatment.

Observation Items	Sep. 25, 2020	Oct. 12, 2020	Nov. 9, 2020	Dec. 11, 2020	Feb. 22, 2021	Apr. 7, 2021	May.17, 2021	Aug. 4, 2021	July. 8, 2022	Dec. 4, 2023
Main Symptoms										
Shortness of breath/Wheezing	6	4	4	4	2	2	2	2	2	2
Fatigue/Weakness	6	4	4	2	2	2	2	2	2	0
Palpitations	6	6	4	4	2	2	2	2	2	2
Chest tightness/Pain	6	4	4	2	2	2	2	2	2	0
Oliguria/Edema	6	4	2	2	2	2	2	2	0	0
Secondary Symptoms										
Symptoms worsen with activity	6	6	4	2	2	2	2	2	2	2
Low voice	4	4	2	2	2	2	0	0	0	0
Spontaneous sweating	6	6	4	2	2	2	0	0	0	2
Cough/Sputum	4	4	2	0	0	0	0	0	0	0
Bloating/Nausea/Vomiting	6	6	6	2	4	0	0	0	0	0
Insomnia	6	6	6	2	2	0	2	0	0	0
Dry mouth/Thirst	2	2	2	4	2	4	2	0	0	0
Cyanotic lips/face	6	6	4	2	2	2	2	2	2	2
Cold intolerance	4	4	2	2	0	0	0	2	2	0
Hot palms/soles	0	0	0	4	2	2	2	2	2	0
Tongue and Pulse										

Swollen tongue, white coating, deep/thin/weak pulse	6	6	6	0	0	2	2	2	2	2
Thin tongue, scant/no coating, fissured/peeled coating, rapid/weak/irregular pulse	0	0	0	4	4	2	2	0	2	2
Total Score	80	72	56	40	32	28	24	20	20	14

Note: None is 0, mild is 2, moderate is 4, severe is 6. As therapeutic interventions advance, there is a corresponding decline in symptom presentation, syndrome scoring metrics, and overall disease burden.
